# Selective genotyping strategies for a sib test scheme of a broiler breeder program

**DOI:** 10.1186/s12711-023-00785-3

**Published:** 2023-03-07

**Authors:** Charlie A. de Hollander, Vivian P. Breen, John Henshall, Fernando B. Lopes, Mario PL. Calus

**Affiliations:** 1 Cobb Vantress, Inc, Siloam Springs, AR USA; 2 Animal Breeding and Genomics, Wageningen University and Research, P.O. Box 338, 6700 AH Wageningen, The Netherlands

## Abstract

**Background:**

In broiler breeding, genotype-by-environment interaction is known to result in a genetic correlation between body weight measured in bio-secure and commercial environments that is substantially less than 1. Thus, measuring body weights on sibs of selection candidates in a commercial environment and genotyping them could increase genetic progress. Using real data, the aim of this study was to evaluate which genotyping strategy and which proportion of sibs placed in the commercial environment should be genotyped to optimize a sib-testing breeding program in broilers. Phenotypic body weight and genomic information were collected on all sibs raised in a commercial environment, which allowed to retrospectively analyze different sampling strategies and genotyping proportions.

**Results:**

Accuracies of genomic estimated breeding values (GEBV) obtained with the different genotyping strategies were assessed by computing their correlation with GEBV obtained when all sibs in the commercial environment were genotyped. Results showed that, compared to random sampling (RND), genotyping sibs with extreme phenotypes (EXT) resulted in higher GEBV accuracy across all genotyping proportions, especially for genotyping proportions of 12.5% or 25%, which resulted in correlations of 0.91 vs 0.88 for 12.5% and 0.94 vs 0.91 for 25% genotyped. Including pedigree on birds with phenotype in the commercial environment that were not genotyped increased accuracy at lower genotyping proportions, especially for the RND strategy (correlations of 0.88 vs 0.65 at 12.5% and 0.91 vs 0.80 at 25%), and a smaller but still substantial increase in accuracy for the EXT strategy (0.91 vs 0.79 for 12.5% and 0.94 vs 0.88 for 25% genotyped). Dispersion bias was virtually absent for RND if 25% or more birds were genotyped. However, GEBV were considerably inflated for EXT, especially when the proportion genotyped was low, which was further exacerbated if the pedigree of non-genotyped sibs was excluded.

**Conclusions:**

When less than 75% of all animals placed in a commercial environment are genotyped, it is recommended to use the EXT strategy, because it yields the highest accuracy. However, caution should be taken when interpreting the resulting GEBV because they will be over-dispersed. When 75% or more of the animals are genotyped, random sampling is recommended because it yields virtually no bias of GEBV and results in similar accuracies as the EXT strategy.

## Background

Broiler poultry breeding companies keep and select their genetic pure lines in a bio-secure environment to reduce the risk of diseases. However, this environment can differ substantially from the environment in which commercial broilers perform, resulting in genotype-by-environment (GxE) interactions. Commercial environments are less sanitary and thus, compared to a bio-secure environment, they can pose different challenges, which affect both the performance and livability of the birds. Genotype-by-environment interactions cause differences in the expression of phenotypes under distinct environments [1–4] and thereby lead to reranking of individuals. Using phenotypes recorded in commercial environments on family members of selection candidates and using these for their genetic evaluation has already been proven to increase genetic gains in economically relevant traits in cattle and pig breeding programs [5–7]. Thus, it is important for a breeding program to quantify the magnitude of the GxE interactions for economically relevant traits. If the genetic correlation between a trait measured in a bio-secure and in a commercial environment is lower than 0.8, efforts should be made to record this trait on families in both environments [7]. It has been reported that the genetic correlation between body weight of the same purebred broiler line recorded in a bio-secure and in a commercial-like environment ranges from 0.46 to 0.69 [8, 9].

Increasing the number of individuals placed in the commercial environment will improve the accuracy of the estimated breeding values (EBV) for a commercial trait and thereby improve the genetic progress [6, 7]. However, there are often restrictions on the number of individuals that can be placed in a commercial environment because of the structure of a broiler breeding program; only a limited number of birds can be hatched per hen in a selection flock, which is a group of eligible selection candidates within a selection round, and only a limited number of hens can be assigned to each rooster, which restricts family sizes. Due to biosecurity rules, birds that are placed in a commercial environment cannot return to a bio-secure environment and can, therefore, not be considered as selection candidates within the nucleus of the breeding program. Thus, although placing a high percentage of birds generated by a selection flock in a commercial environment will improve the accuracy of the EBV for the commercial trait, it can negatively affect selection intensity. It is therefore important to assess what proportion of birds should be tested in the commercial environment to obtain the best balance between accuracy and selection intensity such that genetic gain is optimized.

In a simulation study, Chu et al. [10] found that, with genomic selection, taking selection intensity, inbreeding, and accuracy into account, the proportion of birds to phenotype in a commercial environment to maximize genetic gain for body weight in a commercial environment was 30%. Their results were based on genetic correlations of 0.5 and 0.7 between body weight measured in a bio-secure and in a commercial environment. Chu et al. [8] showed the benefit of using genomic versus pedigree-based prediction when a trait is measured in two environments, which comes from genomic prediction resulting in higher accuracies of EBV because it takes the Mendelian sampling term into account when calculating the relationship between individuals [11, 12]. In our scenario, this is especially useful, as the commercial body weight trait is not recorded on the selection candidates. To balance costs and benefits of genotyping individuals, a breeding company needs to find an optimal genotyping strategy that minimizes costs and maximizes accuracy of EBV, while limiting bias of the EBV used in the breeding program.

Several simulation studies on other species have shown that randomly selecting individuals to be genotyped resulted in higher accuracy and lower bias of genomic EBV (GEBV) for selection candidates compared to strategies where individuals were selected for genotyping based on having the best phenotypes for the traits of interest or having the highest family selection index [13–15]. Other simulations studies found that genotyping based on extreme, i.e. both highest and lowest, family selection indexes or phenotypes resulted in the highest accuracies of GEBV for selection candidates [14–18]. Using real data, the main objectives of this study were: (1) to evaluate which genotype sampling strategy should be used and (2) which proportion of the birds placed in the commercial environment should be genotyped to optimize a broiler breeder sib test scheme.

## Methods

### Phenotypes and genotypes

Data were provided by the poultry breeding company Cobb-Vantress, Inc. (Siloam Springs, Arkansas, USA) and included performance records collected on 24 selection flocks of pure-line broiler chickens that were raised either in a bio-secure environment (Env B) or in a commercial environment (Env C). The birds included in this study were offspring from 519 sires to 2368 dams. About 30% of the birds that hatched within a selection flock were placed in Env C, as recommended by Chu et al. [10]. All these birds were either full- or half-sibs of the remaining 70% birds, which were placed in Env B as selection candidates. For both Env B (BW_B_) and Env C (BW_C_), body weight was recorded at 42 days of age for the first six selection flocks and at 35 days of age for the next 18 selection flocks. In Env B, phenotypes were also recorded on white meat percentage (WMPct), leg quarter percentage (LegQPct), body weight gain (Gain), and feed efficiency (FE). Quality control of the data consisted of removing birds with unknown gender and phenotypes that deviated more than three standard deviations from the average of their contemporary group, which resulted in less than 1% of the data being removed. In addition to gender, the contemporary group of a bird was defined as the interaction between the mating age group of its parents, its own mating age group, and the hatch week it was born. For BW_B_, there were 476 contemporary groups with on average 187 birds per group, and for BW_C_ there were 96, with on average 417 birds per group. Genotypes from a 60 K single nucleotide polymorphism (SNP) panel were collected on all the birds in Env C but only on selection candidates in Env B that were selected based on a selection index. The numbers of birds with phenotypes and genotypes that remained for analyses for each trait are in Table 1. All chicks placed in Env B had a known pedigree.

**Table 1:** Number of birds with phenotypes and genotypes for each trait

	Phenotypes (n)	Genotype (n)
Body weight in a bio-secure environment	87,381	26,867
Body weight in a commercial environment	34,863	34,816
Feed efficiency	30,430	15,992
Breast meat/ leg quarter yield	7606	7594

Only SNPs that fulfilled the following criteria were retained: a call rate higher than 0.90 and a minor allele frequency higher than 0.05. After quality control, 45,993 SNPs remained for analyses. All genotyped birds had a call rate > 0.95, due to quality control criteria for sample delivery.

### Genotyping strategies for Env C

Since all chicks in Env C were genotyped, we were able to retrospectively evaluate different genotyping strategies and proportions. Three strategies were used to select chicks in Env C for genotyping:

1. Genotyping of randomly selected birds (RND).
2. Genotyping of randomly selected birds within sire families (SIRE).
3. Genotyping of birds with extreme phenotypes, i.e. those with the highest and lowest body weight (EXT).

**Fig. 1:**
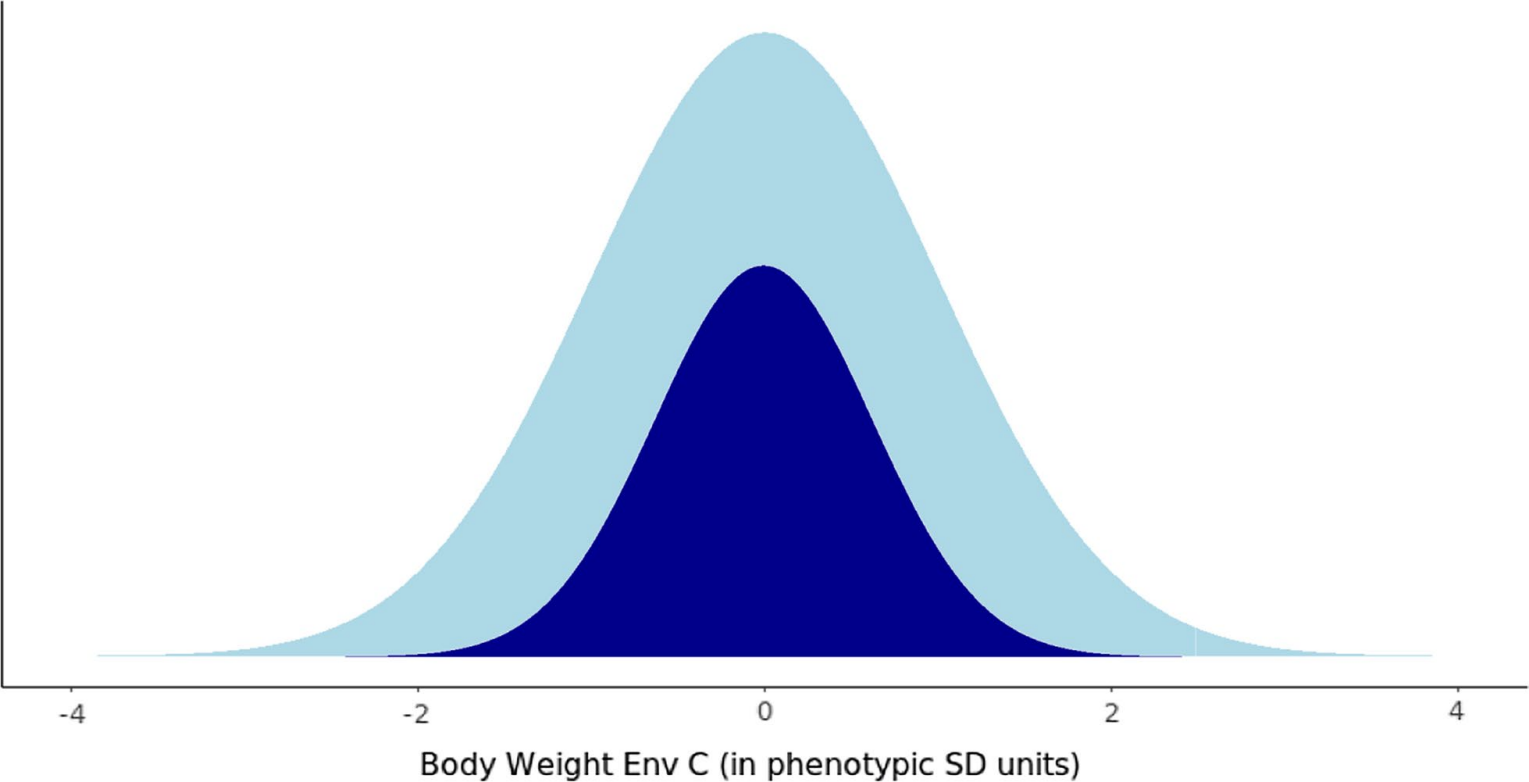
Illustration of the full distribution of body weights measured in the commercial environment (denoted in light Blue), and the distribution of body weights of birds sampled by the Random and Sire genotyping sampling strategy (denoted in Dark Blue)

For each genotyping strategy, results were evaluated for four proportions of birds genotyped: 12.5, 25, 50, and 75%. In the SIRE strategy these proportions were applied within each sire family, while in the EXT scenario using a genotyping proportion of e.g. 25% involved genotyping the 12.5% highest and the 12.5% lowest body weight animals. The number of genotyped birds always remained the same in Env B. Because birds were randomly sampled in the RND and SIRE strategies, in these strategies the body weights of the genotyped individuals are still expected to follow a normal distribution, as illustrated in Fig. 1.

**Fig. 2:**
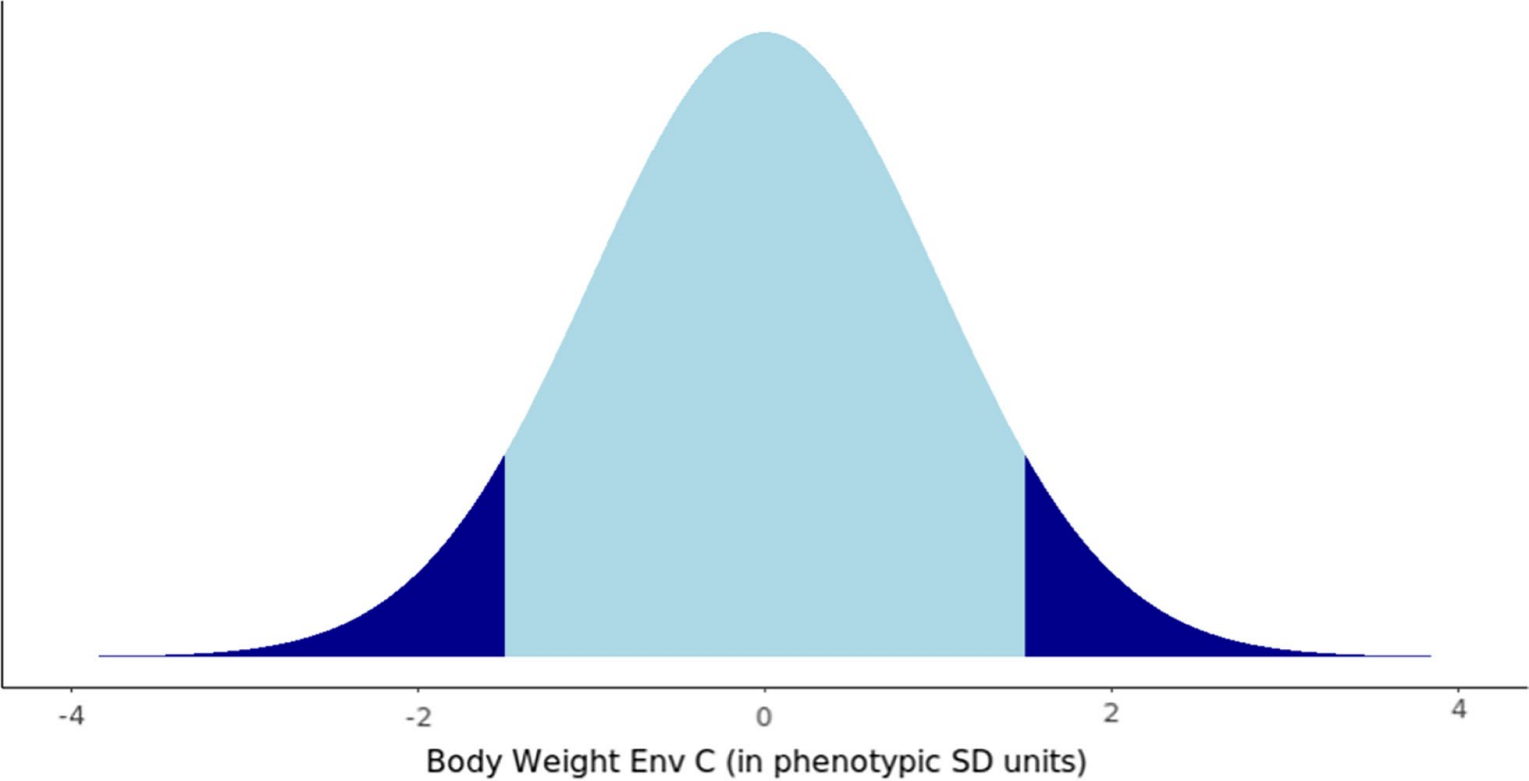
Illustration of the extreme phenotype genotyping sampling strategy in which birds from the tail ends of the normal distribution for body weight measured in the commercial environment are genotyped (denoted in Dark Blue)

For the EXT scenario, half of the genotyped birds were the top ranking and the other half were the bottom ranking birds based on phenotype for body weight. Thus, in the EXT scenario, birds from the tail ends of the distribution of body weight were genotyped, as illustrated in Fig. 2. The average number of genotyped birds used for each proportion genotyped over the last six selection flocks is shown in Table 2.

**Table 2:** Average number of genotyped birds in Env C for the different proportions of birds genotyped over the six selection flocks

	Proportion genotyped in Env C				
	12.5%	25%	50%	75%	100%
Number of genotyped birds	3460	6936	13,886	20,834	27,793

For the RND and EXT genotyping strategies, analyses with and without pedigree information on non-genotyped birds were carried out to reflect scenarios in which the chicks placed in Env C are hatched in a selection hatchery, in which case their pedigree would be known, or in a commercial hatchery, in which case their pedigree would not be known. The SIRE sampling strategy requires that the pedigree of the chicks is known and, therefore, analysis for this strategy was done with availability of pedigree information only.

### Statistical analyses

#### Models

A multivariate pedigree-based model was used to estimate variance and covariance components for all six traits, using the full dataset. When only a proportion of the animals is selectively genotyped, it is recommended to use a pedigree-based animal model to estimate the variance components because it yields less biased variance components than a single-step model that includes genomic information [19, 20]. For BW_B_ and BW_C_, a random permanent maternal environmental effect was added to the model because early body weight is affected by maternal effects, for example through egg size [21, 22]. The model for BW_B_ and BW_C_ was:

$$\mathbf{y} = \mathbf{Xb} + \mathbf{Za} + \mathbf{Wc} +\mathbf{e},$$

And the model for Gain, FE, WMPct% and LegQ% was:

$$\mathbf{y} = \mathbf{Xb} + \mathbf{Za} +\mathbf{e},$$

where $\mathbf{X}$, $\mathbf{Z},$ and $\mathbf{W}$ are incidence matrices; $\mathbf{b}$ is a vector of the fixed effects of contemporary group, and $\mathbf{a}$, $\mathbf{c}$**,** and $\mathbf{e}$ are vectors of direct additive genetic, permanent maternal environmental, and residual effects, respectively. These random effects were assumed to be normally distributed $\mathbf{a} \sim N(\mathbf{0},\mathbf{A}\otimes {\mathbf{G}}_{{\varvec{a}}}$), $\mathbf{c} \sim N(\mathbf{0},{\mathbf{I}}_{\mathbf{d}}\otimes \mathbf{C}$), and $\mathbf{e} \sim N(\mathbf{0},\mathbf{I}\otimes \mathbf{R})$, where $\mathbf{A}$ is the pedigree relationship matrix, ${\mathbf{I}}_{\mathbf{d}}$ and $\mathbf{I}$ are identity matrices, ${\mathbf{G}}_{{\varvec{a}}}$ is the additive genetic covariance matrix among traits, $\mathbf{C}$ is the permanent maternal environmental covariance matrix, and $\mathbf{R}$ is the residual covariance matrix. This model has been used in several other studies [9, 23, 24] for body weight measured at similar ages as used here. Variance components were estimated by the AIREML procedure in the DMUAI module of the DMU software [25]. Standard errors (SE) of the estimates of heritability and genetic correlations were approximated using Taylor series expansions, as described in [25, 26].

Resulting estimates of variance components were subsequently used in a single step genomic best linear unbiased prediction (ssGBLUP) model, implemented with the BLUPF90 software [27], which combines pedigree and genomic information to predict GEBV [28–30]. The models used for ssGBLUP were identical to those used to estimate genetic parameters, except that the inverse of the pedigree relationship matrix (${\mathbf{A}}^{-1}$) was replaced by the inverse of the combined relationship matrix (${\mathbf{H}}^{-1}$) [28, 30], which was constructed by combining ${\mathbf{A}}^{-1}$, the inverse of the pedigree-based relationship matrix of the genotyped animals (${\mathbf{A}}_{22}^{-1}$), and the inverse of the genomic relationship matrix ${\mathbf{G}}^{-1}$. To do so, a blended $\mathbf{G}$ was used and was defined as:

$$\mathbf{G}=0.95{\mathbf{G}}_{\mathbf{0}}+0.05{\mathbf{A}}_{22},$$

where ${\mathbf{G}}_{\mathbf{0}}$ is the genomic relationship matrix based on method 1 of VanRaden [31]. In addition, inbreeding was accounted for in ${\mathbf{G}}^{*}$, by matching the relationship matrix $\mathbf{G}$ to the relationship matrix ${\mathbf{A}}_{22}$. This was done as:

$${\mathbf{G}}^{*} =\left(1-\frac{\rho }{2}\right)\mathbf{G}+{\mathbf{11}^{\prime}}\rho ,$$

where $\rho$ is the adjustment factor proposed by Vitezica et al. [32].

Thereafter the matrix ${\mathbf{H}}^{-1}$ was constructed as:

$${\mathbf{H}}^{-1}={\mathbf{A}}^{-1}+ \left[\begin{array}{cc}0& 0\\ 0& {\mathbf{G}}^{*-1}-{\mathbf{A}}_{22}^{-1}\end{array}\right].$$

As ${\mathbf{G}}^{*-1}$ becomes more of a computational burden when the number of genotypes increases, it was decided to calculate the inverse of the ${\mathbf{G}}^{*}$ matrix by using the algorithm for proven and young (APY) [33], using all parents of the animals with phenotypes in the dataset, and all selection candidates up to three generations in the past as core animals (c) and the rest as non-core animals (n). Thus, ${\mathbf{G}}^{*}$ was partitioned as:

$${\mathbf{G}}^{*}=\left[\begin{array}{cc}{\mathbf{G}}_{\mathbf{cc}}& {\mathbf{G}}_{\mathbf{cn}}\\ {\mathbf{G}}_{\mathbf{nc}}& {\mathbf{G}}_{\mathbf{nn}}\end{array}\right],$$

where ${\mathbf{G}}_{\mathbf{cc}}$, ${\mathbf{G}}_{\mathbf{nc}}$, ${\mathbf{G}}_{\mathbf{c}\mathbf{n}}$ as well as the diagonal elements of ${\mathbf{G}}_{\mathbf{nn}}$ were computed and used to construct the APY inverse [33].

The number of core and non-core animals for each genotyping proportion are in Table 3. Initial analyses showed that the GEBV obtained with APY were virtually the same as those obtained using the full $\mathbf{G}$*^−1^ matrix.

**Table 3:** Average number of core and non-core animals per selection round for each scenario

	Proportions genotyped in Env C			
	12.5%	25%	50%	75%
Average number of core animals	6482	7251	8777	10,300
Average number of non-core animals	17,412	20,119	25,544	30,967

### Validation

Validation was done using the LR method [34] to evaluate the differences in GEBV accuracies and dispersion bias. This validation method assumes that GEBV computed using full data are more accurate than those based on partial data, where “full” refers to a given dataset and “partial” simply means that only a part of the full data is used. Each of the last six selection flocks (19 to 24) was used in turn as validation population and the data from the 18 selection flocks that immediately preceded the validation flock was used as training data. Thus, when selection flock 19 was used for validation, data from selection flocks 1 to 18 were included in the analysis, and when selection flock 20 was used as validation, data from selection flocks 2 to 19 were included in the analysis, and so on. The reduction in accuracy of each genotyping strategy was then measured as the correlation (${r}_{f-r}$) of GEBV from birds in the validation selection flock obtained in this genotyping strategy with their GEBV obtained from the full data. Values of ${r}_{f-r}$ close to 1 suggest that the corresponding genotyping strategy achieved similar GEBV and, therefore, similar accuracies, as obtained when using the full data. The full dataset contained the genotypes of all birds raised in Env C and the reduced dataset only contained a proportion of the genotyped birds raised in Env C based on each genotyping strategy. All selection candidates in Env B had genotypes and the number of animals with genotypes was the same for all strategies as in the full data. Dispersion bias of GEBV for the selection candidates under each genotyping strategy was evaluated by the slope of the regression of the GEBV from the full data on the GEBV of the reduced data for each sampling strategy. All results are presented as averages across the six validation selection flocks. The SE for the correlations and regression coefficients between the full and reduced data across the six validation selection flocks were calculated as the standard deviation of the six estimates divided by the square root of 6.

## Results and discussion

### Genetic parameters

Estimates of heritabilities for all traits and of the genetic correlations between the traits are in Table 4. The estimate of heritability was slightly lower for BW_B_ than for BW_C_. Estimates of both the additive genetic and residual variance were larger for BW_c_, but the difference in residual variance estimates was proportionally smaller. The heritability estimates for BW_B_ and BW_C_ confirmed those reported by Chu et al. [8], who found heritabilities of 0.31 to 0.37 for body weight recorded in Env B and Env C for older generations of the populations used here. The estimate of the genetic correlation of BW_B_ with BW_C_ was 0.59, which indicates presence of GxE interaction. This result is in agreement with those of Kapell et al. [9] and Chu et al. [8], who reported a genetic correlation of 0.46 to 0.69 between body weight measured on a bio-secure and on a commercial farm. Estimates of genetic correlations of the two dissection traits, WMPct and LegQPct, were lower with BW_C_ than with BW_B_, which is as expected because BW_C_ is measured in a different environment. Estimates of the genetic correlation of feed efficiency were close to zero with both BW_B_ and BW_C_, because FE was corrected for body weight. Since the estimate of the genetic correlation between BW_B_ and BW_C_ was considerably less than 1 (0.59) and the estimate of the heritability for BW_C_ was moderately high (0.37), setting up a sib test scheme is expected to increase genetic gain in body weight in a commercial environment [7, 10].

**Table 4:** Estimates of heritabilities and standard errors (SE) (in bold on the diagonal) and of genetic correlations (below the diagonal) and their standard errors (above the diagonal)

	BW_B_	BW_C_	WMPct	LegQPct	FE	Gain
BW_B_	**0.34 (0.02)**	0.04	0.05	0.05	0.04	0.04
BW_C_	0.59	**0.37 (0.03)**	0.05	0.05	0.05	0.05
WMPct	0.25	0.04	**0.61 (0.03)**	0.05	0.05	0.05
LegQPct	− 0.06	0.02	− 0.39	**0.60 (0.03)**	0.05	0.05
FE	0.02	0.03	− 0.26	0.08	**0.24 (0.01)**	0.05
Gain	0.30	0.11	0.02	0.01	0.18	**0.22 (0.01)**

Bold characters refer to the esstimates of heritabilities and their standard errors (SE)

*BW*_*B*_ body weight recorded in Env B; *BW*_*C*_ body weight recorded in Env C; *WMPct* white meat percentage; *LegQPct* leg quarter percentage; *FE* feed efficiency; and Gain: bodyweight gain

### Accuracy

The correlation of the EBV of the validation selection flocks for BW_C_ when using pedigree only and GEBV obtained with all genotypes included was 0.83 (Table 5).

**Table 5:** Correlations (${{\varvec{r}}}_{{\varvec{f}}-{\varvec{r}}}$) of EBV for BW_C_ of birds from the six validation selection flocks obtained from the reduced data with EBV obtained from the full data and their standard error (SE) for different genotyping strategies and proportions genotyped and with or without availability of pedigree on non-genotyped animals in the commercial environment

Genotyping strategy	Proportion of genotyped animals in Env C (%)	${r}_{f-r}$	SE
Pedigree only	0.00	0.83	0.004
RND_Ped_	12.50	0.88	0.003
RND_NoPed_	12.50	0.65	0.013
SIRE	12.50	0.88	0.002
EXT_Ped_	12.50	0.91	0.003
EXT_NoPed_	12.50	0.79	0.012
RND_Ped_	25	0.91	0.003
RND_NoPed_	25	0.80	0.007
SIRE	25	0.91	0.003
EXT_Ped_	25	0.94	0.002
EXT_NoPed_	25	0.88	0.004
RND_Ped_	50	0.95	0.002
RND_NoPed_	50	0.89	0.004
SIRE	50	0.95	0.003
EXT_Ped_	50	0.97	0.001
EXT_NoPed_	50	0.94	0.002
RND_Ped_	75	0.98	0.001
RND_NoPed_	75	0.95	0.002
SIRE	75	0.98	0.001
EXT_Ped_	75	0.99	0.000
EXT_NoPed_	75	0.97	0.001

*RND*_*Ped*_ pedigree available on all birds in Env C, genotypes are sampled randomly; *RND*_*NoPed*_ pedigree is only available on genotyped birds, genotypes are sampled randomly; *SIRE* pedigree available on all birds, genotypes are sampled randomly within sire family; *EXT*_*Ped*_ pedigree available on all birds, genotypes are sampled based on extreme phenotypes; *EXT*_*NoPed*_ pedigree is only available on genotyped birds, genotypes are sampled based on extreme phenotypes

Thus, in addition to having pedigree information, genotyping birds with phenotypes in the commercial environment is beneficial in a sib test scheme for broilers, as already reported by Chu et al. [8] for similar data. For all genotyping strategies, ${r}_{f-r}$ increased as the number of genotyped birds increased, which is in agreement with previous simulation studies [11, 14, 15, 18, 35, 36]. For a given genotyping proportion, ${r}_{f-r}$ was highest for the EXT strategy, which suggests that this strategy achieved the highest accuracy. Previous simulation studies also found that accuracies were higher when individuals with extreme phenotypes, i.e. the highest and lowest, were genotyped instead of genotyping only individuals with the highest family index or highest performance [14–18, 37]. Genotyping of animals with extreme phenotypes is more informative for prediction of Mendelian deviations from the mid parent mean [37], which is especially useful when the trait of interest is not recorded on the selection candidates. In addition, Boligon et al. [16] showed that genotyping animals with extreme phenotypic deviations for an indicator trait that has a genetic correlation of 0.5 with the trait of interest, provided the highest accuracy of GEBV for the trait of interest in the next generation. This suggests that it may be useful to also genotype animals with low BW_B_, in addition to the selection candidates, to further increase the accuracy of EBV for selection candidates for BW_C_. However, since genotyping is costly and birds that are not yet genotyped for the purpose of the breeding program are not selection candidates, this strategy may not be justified in practice.

The results in Table 5 demonstrate that there is little difference between the RND_Ped_ and SIRE strategies, as both show a very high accuracy when 50% or more of the birds are genotyped. When more than 50% of birds are genotyped, both the RND_Ped_ and SIRE strategies represent a random and unbiased amount of phenotypic variation across the population and provide a good representation of the families, whereas selective genotyping based on the best family index or phenotype, will result in lower family representation or phenotypic variation and, therefore, in lower accuracies. This is consistent with previous simulation studies that showed that random selective genotyping results in higher accuracies than selective genotyping based on family index or phenotypes [13–18]. For all strategies, sampling 75% of all birds led to GEBV that were very similar to those based on the full data (${r}_{f-r}$=0.95–0.99). The RND_Ped_, SIRE and EXT_Ped_ strategies achieve high accuracies compared to the full data even when 50% of the birds are genotyped (0.95, 0.95 vs 0.97, respectively). Thus, genotyping 100% of the birds might not be necessary and reducing the number of genotyped animals can help minimize costs of the breeding program. The results also suggest that accuracy considerably increased when pedigree information is available for the non-genotyped birds, especially when sampling proportions are small, i.e. with 12.5% genotyped, ${r}_{f-r}$ increased from 0.65 to 0.88 for RND and from 0.79 to 0.91 for EXT, and from 0.80 to 0.97 for RND and from 0.88 to 0.94 for EXT with 25% genotyped. However, with 75% genotyped, the drop in accuracy due to the lack of pedigree information was small for both RND_NoPed_ and EXT_NoPed_ (${r}_{f-r}$ of 0.95 vs 0.97). Thus, hatching chicks in a commercial hatchery in a sib scheme test as mimicked by RND_NoPed_ and EXT_NoPed_ is a valid option, provided the proportion of birds genotyped is sufficiently high (i.e. 50% or more).

It should also be noted that the estimates of ${r}_{f-r}$ for all but one trait (WMPct, LegQPct, FE, and Gain) were not affected by the number of birds genotyped in Env C (results not shown). This is because the number of records for these traits was the same across all strategies and proportions of birds genotyped in Env C. These results suggest that, regardless of the genotyping strategy or the proportion of birds genotyped, the accuracy of GEBV for traits measured in the bio-secure environment is not affected by the genotyping strategy or the proportion of birds being genotyped in Env C.

### Dispersion bias

Although prediction bias does not lead to reranking of animals with single trait selection, it will affect selection decisions and index weighting by deflating or inflating EBV across generations. This makes interpretation of genetic trends unreliable and comparisons between animals across generations difficult [38, 39]. Thus, bias of EBV should be minimized as much as possible. If the regression slope is smaller than 1 (β < 1), GEBV are over-dispersed and response to selection is overestimated. If regression slopes are greater than 1 (β > 1), GEBV are under-dispersed and response to selection is underestimated. The dispersion bias of BW_C_, computed under the assumption that the GEBV based on the full data are unbiased, is shown in Table 6. When the proportion of genotyped animals is low, the GEBV are clearly over dispersed, i.e. β < 1.

**Table 6:** Regression coefficients (β_f-r_) of GEBV for BW_C_ based on the full data on the GEBV based on the reduced data for each genotyping strategy

Genotyping strategy	Proportion genotyped (%)	β_f-r_	SE
Pedigree only	0.00	0.88	0.012
RND_Ped_	12.50	0.90	0.008
RND_NoPed_	12.50	0.80	0.020
SIRE	12.50	0.90	0.005
EXT_Ped_	12.50	0.75	0.008
EXT_NoPed_	12.50	0.47	0.008
RND_Ped_	25	0.95	0.010
RND_NoPed_	25	0.98	0.018
SIRE	25	0.93	0.009
EXT_Ped_	25	0.78	0.007
EXT_NoPed_	25	0.58	0.007
RND_Ped_	50	0.96	0.006
RND_NoPed_	50	0.98	0.017
SIRE	50	0.95	0.006
EXT_Ped_	50	0.85	0.006
EXT_NoPed_	50	0.76	0.010
RND_Ped_	75	1.00	0.008
RND_NoPed_	75	1.01	0.016
SIRE	75	0.99	0.002
EXT_Ped_	75	0.93	0.003
EXT_NoPed_	75	0.91	0.008

*RND*_*Ped*_ pedigree available on all birds in Env C, genotypes are sampled randomly; *RND*_*NoPed*_ pedigree is only available on genotyped birds, genotypes are sampled randomly; *SIRE* pedigree available on all birds, genotypes are sampled randomly within a sire family; *EXT*_*Ped*_ pedigree available on all birds, genotypes are sampled based on extreme phenotypes; *EXT*_*NoPed*_ pedigree is only available on genotyped birds, genotypes are sampled based on extreme phenotypes

Since for both the RND_ped_ and SIRE strategies genotyped animals were sampled randomly, while pedigree was considered to be available for all birds in Env C, we would expect to see no dispersion bias for those strategies. Nevertheless, for those strategies β did decrease with decreasing proportion genotyped, with a lowest value of 0.88 if no genotypes were available at all for Env C. This may suggest that in spite of the adjustment of the genomic relationship matrix to match the pedigree relationship matrix, there were still some inconsistencies between both matrices. The dispersion bias was largest with the EXT strategy compared to the RND and SIRE strategies across all genotyping proportions and with or without pedigree information. Many simulation studies have reported large biases when only genotyping animals with extreme phenotypes [15, 18], especially when the proportion of genotyped animals is low. When the number of genotyped animals increases, the dispersion bias gradually disappeared, i.e. β approached 1. The latter agrees with observations from several other studies [11, 15, 35], and can be explained by the fact that, with a larger proportion animals genotyped, they provide a better representation of the entire population. Dispersion bias in a breeding program due to a limited number of genotyped animals is partly overcome by including all non-genotyped animals in ssGBLUP [28–30]. Our results show the benefit of including the pedigree of the ungenotyped animals, especially when the EXT strategy was used. The EXT_NoPed_ strategy showed greater dispersion bias than the EXT_Ped_ strategy for almost all genotyping proportions: 0.47 vs 0.75 at 12.5%, 0.58 vs 0.78 at 25%, and 0.76 vs 0.86 at 50%. In contrast, a notable difference in bias was only observed for RND_Ped_ and RND_NoPed_ at 12.5% (0.81 vs 0.90). No difference in dispersion bias was detected between the RND and SIRE strategies, and bias was virtually absent for both strategies when 25% or more birds were genotyped. Thus, a random genotyping strategy does not have to aim for all sires to be represented, which is in agreement with results of several simulation studies that have shown that bias was smaller with random genotyping compared to selective genotyping [14–17, 37]. Finally, violation of the assumption that is made here, i.e. that there is no dispersion bias in GEBV calculated with the full data, may affect the interpretation of our results. As discussed above, the patterns observed in our results align well with our expectations and with results of other studies, suggesting that indeed there was no or very little bias present in the full data GEBV.

## Conclusions

For a broiler sib test scheme in a commercial environment that results in siblings being removed from the breeding program, we recommend the following genotyping strategies when the correlation between body weights measured in the bio-secure versus commercial environments is  ~ 0.6: (1) when less than 75% of all animals tested in the commercial environment are genotyped, animals with extreme phenotypes should be genotyped since this yields the highest accuracy of GEBV for body weight in the commercial environment; however, the GEBV should be interpreted with caution since they are over dispersed, especially when a limited number of genotyped birds is available in each generation; and (2) when the proportion of genotyped animals is 75% or more, animals should be randomly sampled for genotyping since this reduces the bias and yields accuracies that are similar to those obtained when using the high and low ranked animals based on body weight.

## Data Availability

The data used in the present study were provided by Cobb Vantress, Inc. and are not publicly accessible.
